# Supplementary material for: The importance of meal timing for maintenance of daily rhythms in the gut transcriptome and microbiota
Source: NPJ Biol Timing Sleep. 2026 Jun 22;3:29. doi: 10.1038/s44323-026-00089-x (PMC13287471; doi:10.1038/s44323-026-00089-x)
Supplement: Supplementary file 1 — Supplementary figures [file 44323_2026_89_MOESM1_ESM.pdf]

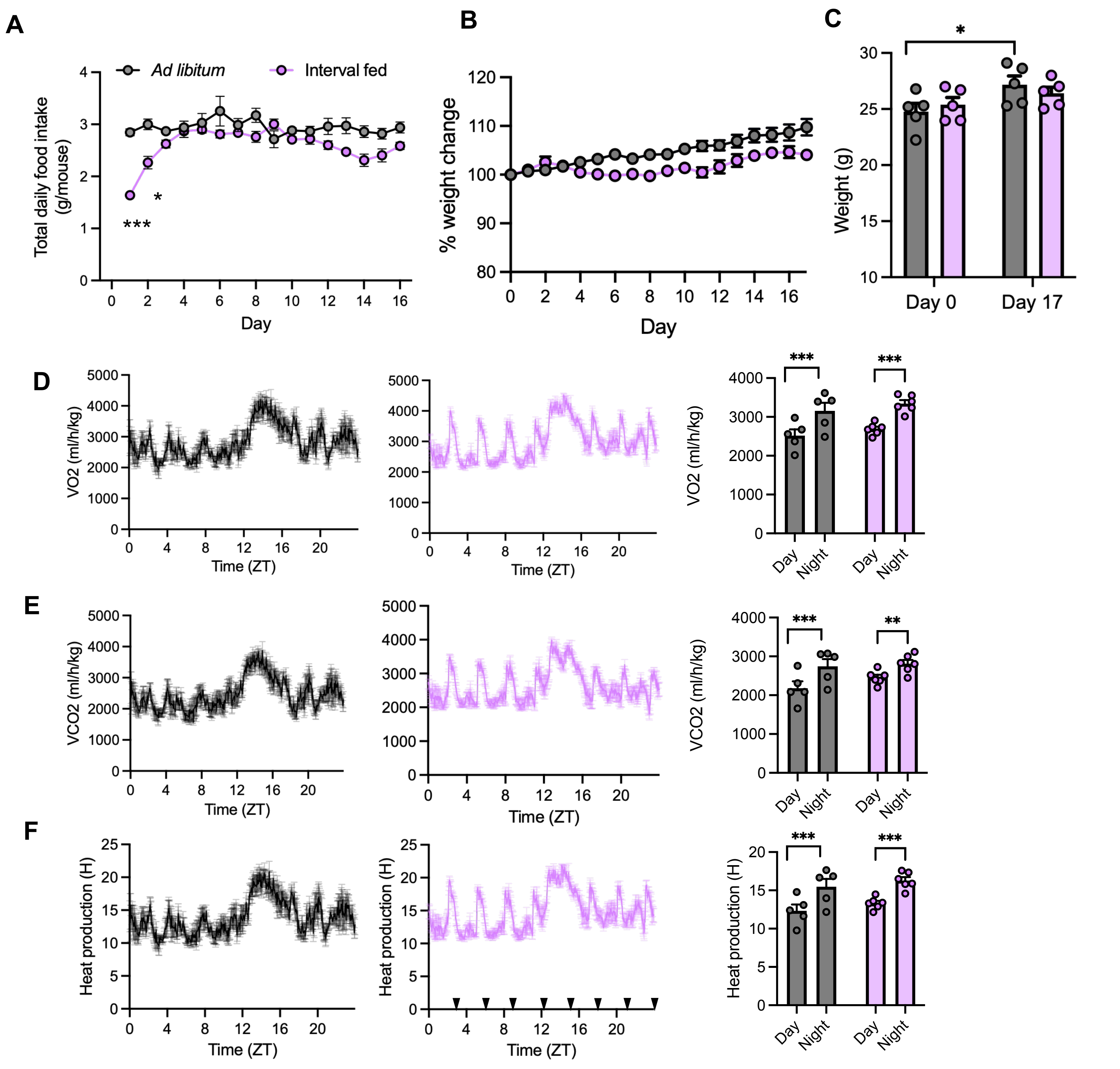

Supplementary Figure 1: (A) Total daily food intake (g) per mouse across feeding regimen, n=15 mice (*ad libitum*) and 30 mice (interval fed). 2-way repeated measures ANOVA and post-hoc Sidak's multiple comparisons test. (B) Weight change across feeding regimen n=5/group, 2-way repeated measures ANOVA and post-hoc Sidak's multiple comparison tests, all NS. (C) Animal weights across feeding regimen at the start (day 0) and end (day 17), n=5/group, 2-way ANOVA and post hoc Sidak's multiple comparisons test. (D) VO2 (E) VCO2 and (F) Heat production under *ad libitum* feeding (black) and interval feeding (purple) as measured in Phenomaster cages. Line plots show group average (n=6 interval fed, n=5 *ad libitum* fed). Bar charts quantify partitioning into day (12h lights on) and night (12h lights off), 2 way ANOVA and post-hoc Tukey's multiple comparisons test.

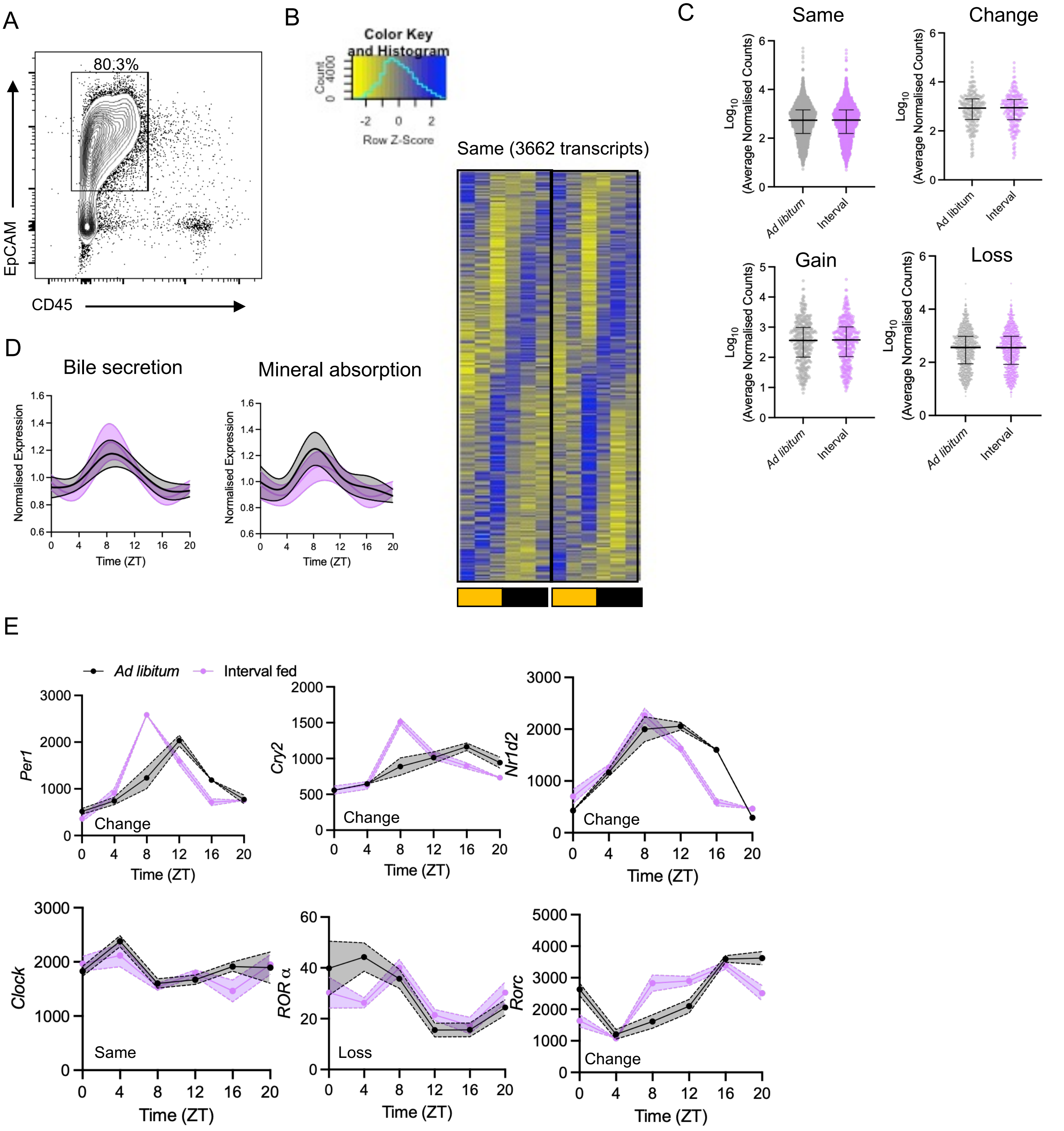

Supplementary Figure 2: (A) Flow cytometric analysis of IECs (EpCAM$^+$ CD45$^-$) harvested from the colon, representative of n=3. (B) Transcripts demonstrating no change in rhythmicity ("same") under interval feeding, n=4-5/timepoint, 6 time points per condition. (C) Mean expression levels (across all time points) of transcripts assigned same, change, gain or loss compared in *ad libitum* and interval fed samples, error bars show median and interquartile range, distributions compared by Welche's t test, all ns. (D) Spline plots of normalised expression of all genes in the "bile secretion" (Mmu_04976, 50 genes) and "Mineral absorption (Mmu_04978, 37 genes) pathways which appeared in our data, error bars represent 95% CIs around the mean. (E) Expression of clock genes within IECs (normalised expression) over time and their rhythmicity as assigned by CompareRhythms, n=4-5/time point.

Example transcripts associated with steroid biosynthesis pathway (KEGG 2019 database)

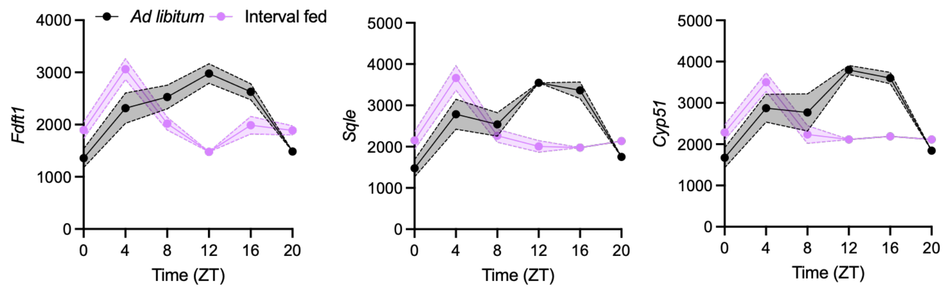

Example transcripts associated with bile secretion pathway (KEGG 2019 database)

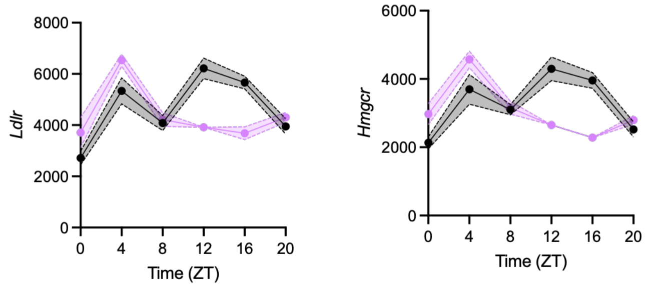

Example transcripts associated with mineral absorption pathway (KEGG 2019 database)

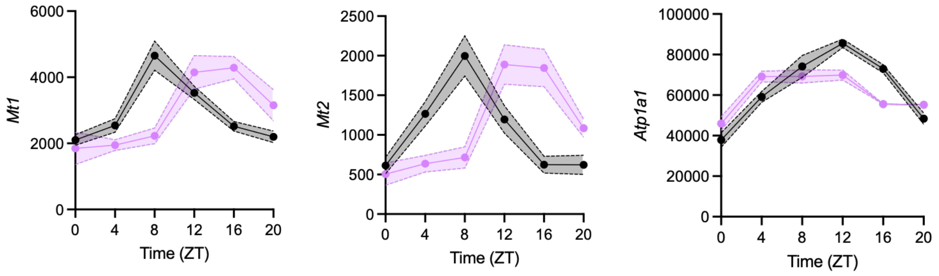

Supplementary Figure 3: Examples of transcripts which were determined to be changed in rhythmic expression (CompareRhythms analysis) in response to interval feeding within the IEC (normalised expression), n=4-5/time point.

Liver clock genes

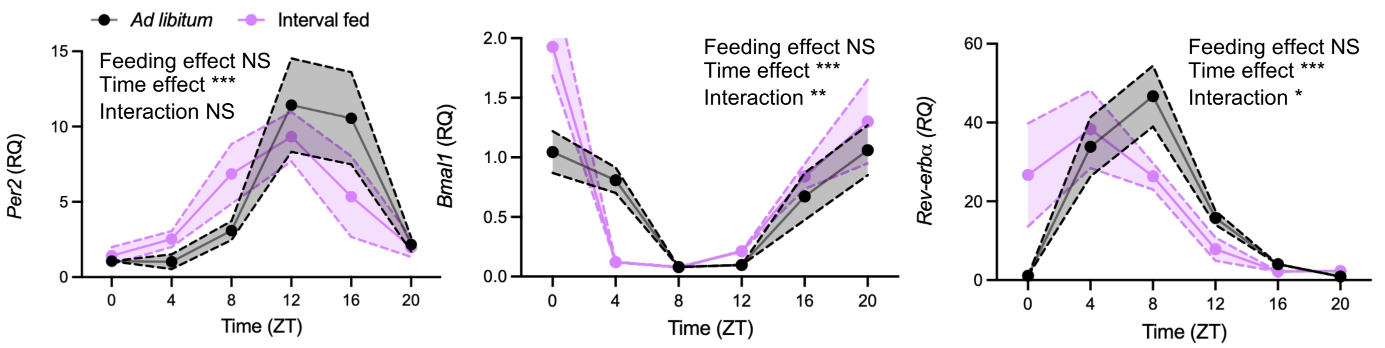

Supplementary Figure 4: Expression of core clock genes (*Per2, Bmal1* and *Rev-erb*α) over time within the liver of *ad libitum* and interval fed mice. Target gene expression was normalised to β-actin and expressed relative to ZT0 in *ad libitum* fed mice. Values are mean±SEM, n=3-5, 2 Way ANOVA, first level significance reported.

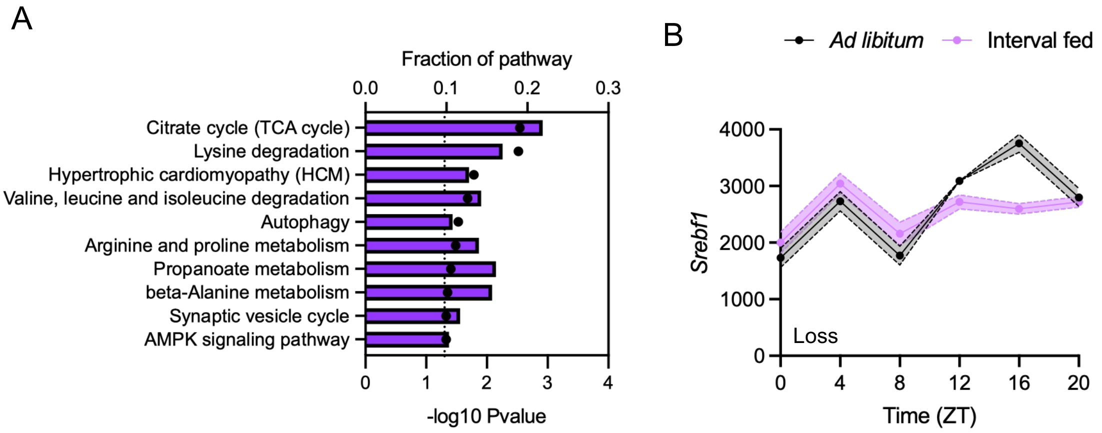

Supplementary Figure 5: (A) Pathway analysis (mouse KEGG 2019) of transcripts which lost rhythmicity under interval feeding. Bars quantify fraction of the pathway represented, and dots represent statistical significance (dotted line marks P=0.05). (B) Expression of *Srepb1* across time in colonic IECs, 24h rhythmicity was lost in interval fed mice (CompareRhythms analysis), n=4-5/time point.

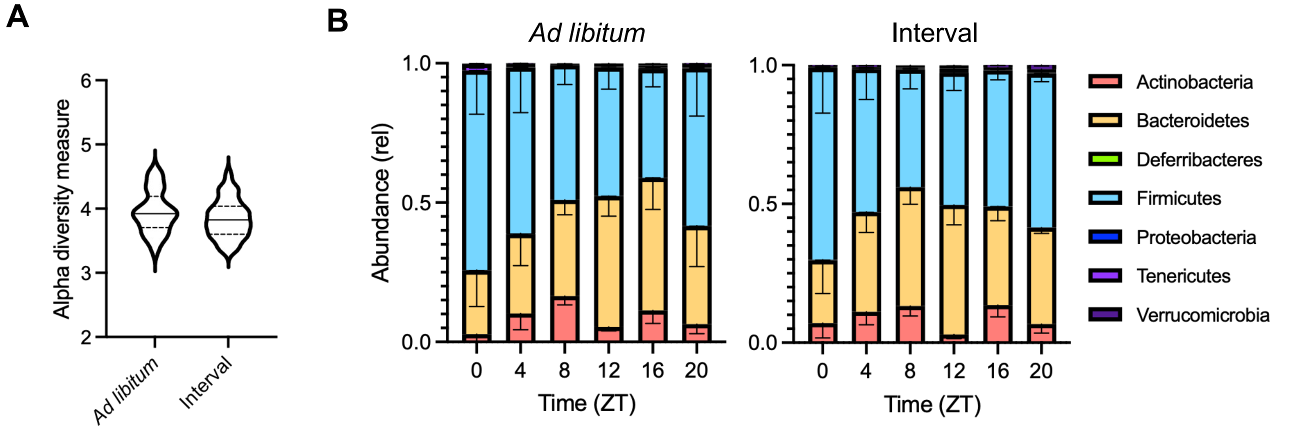

Supplementary Figure 6: (A) Violin plot showing alpha diversity (Shannon Index) between feeding regimens, solid horizontal lines represent group median and dashed horizontal lines represent quartiles, n=30, unpaired T test not significant. (B) Relative abundance by phyla across time, n=5/timepoint.

SCFA	*Ad libitum*		Interval Fed		2 Way ANOVA
	BH.Q	LAG	BH.Q	LAG	
Acetic acid	0.261	-	1.0	-	Feeding NS<br>Time NS<br>Interaction NS
Butyrate	0.204	-	1.0	-	Feeding NS<br>Time NS<br>Interaction NS
Caproic acid	0.022	20	1.0	-	Feeding NS<br>Time *<br>Interaction NS
Proprionic acid	0.283	-	1.0	-	Feeding NS<br>Time NS<br>Interaction NS
Valeric acid	0.036	22	1.0	-	Feeding NS<br>Time *<br>Interaction NS

Supplementary Table 1: Caecal short chain fatty acids (SCFAs) under *ad libitum* and interval feeding. JTK_Cycle analysis of 24h periodicity under each feeding condition and between group comparisons using 2 Way ANOVA.

Gene	Probe (FAM-TAMRA)	Forward	Reverse
B-actin	TGC CAC AGG ATT CCA TAC CCA AGA AGG	AGG TCA TCA CTA TTG GCA ACG A	CAC TTC ATG ATG GAA TTG AAT GTA GTT
Bmal1	TGA CCC TCA TGG AAG GTT AGA ATA TGC AGA A	CCA AGA AAG TAT GGA CAC AGA CAA A	GCA TTC TTG ATC CTT CCT TGG T
Per2	ACT GCT CAC TAC TGC AGC CGC TCG T	GCC TTC AGA CTC ATG ATG ACA GA	TTT GTG TGC GTC AGC TTT GG
Dbp	TGA ACC TGA TCC GGC TGA TCT TGC C	CCG TGG AGG TGC TAA TGA CCT	CCT CTG AGA AGC GGT GCC T
Nr1d1	Commercial primer probe set (Mm00520708) from Applied Biosystems		

Supplementary Table 2: QPCR primers and probes

Supplementary Data Files

Supplementary Data 1: Compare Rhythms analysis. Output from Compare Rhythms analysis of RNASeq of colonic intestinal epithelial cells. Normalised expression across time of each gene in *ad libitum* (AL) and interval fed (IF) animals displayed with amplitude (amp), phase and weights.

Supplementary Data 2: Spline plots data. Normalised expression across time of each gene contributing to spline plots. IF, interval fed and ad lib, *ad libitum* fed. N=4-5/condition/time point.

Supplementary Data 3: JTK_Cycle output. Relative expression of operational taxonomic units (OTUs) across time in *ad libitum* fed and interval fed animals with outputs from JTK_cycle analysis (BH.Q, ADJ.P, Period (PER), lag and amplitude (AMP)) and taxonomic mapping of OTUs. N=5/condition/timepoint.
